# Effect of biochar on biochemical properties of saline soil and growth of rice

**DOI:** 10.1016/j.heliyon.2023.e23859

**Published:** 2023-12-30

**Authors:** Shuqian Zhang, Lingyue Wang, Junping Gao, Baichuan Zhou, Wei Hao, Di Feng, Xiaoan Sun

**Affiliations:** Weifang University of Science and Technology, Shouguang, Shandong, 262700, China

**Keywords:** Biochar, Rice growth, Saline soil, Soil biochemical properties, Yield

## Abstract

To amend physical properties of coastal saline soil for rice production, six biochar treatments (0, 0.5, 1, 2, 4 and 6 kg biochar per m^2^ soil) were set up as CK, T1, T2, T3, T4 and T5, respectively and their effect on the biochemical properties of coastal saline soil and rice growth characteristics were evaluated in a barrel planting experiment. The results showed that compared with CK (with no biochar added), the soil EC of T1 and T2-T5 was reduced by 11.5 %, but increased by 8.8–62.9 %, respectively. The available potassium and organic matter contents of T1-T5 increased ranging from 3.7-10.2 % to 8.0–46.8 %, respectively. With the increase of the biochar amount, the urease activity of soil in the 0–10 cm deep soil showed an increasing trend by 194.8–744.6 % with T1-T5, compared with that of the CK treatment. The activity of alkaline phosphatase in soil increased first and then decreased, and its increment with T1-T5 was between 28.2 and 64.8 % in comparison with that of CK. With more biochar added to soil, the leaf dry weight, root dry weight, total dry matter mass, total root length, single panicle quality and weight per 100 grains showed a trend of increase first and then decrease. The highest incremental values of all measurements were obtained with T1 by 21.8 %, 23.9 %, 13.8 %, 33.9 %, 30.8 % and 11.6 % respectively, compared with those with CK. However, adding biochar in soil demonstrated insignificant effect on the weight of single panicle, panicle length, stem thickness, tillers, setting rate, soil hydrolyzable nitrogen, available phosphorus content, rice protein, amylose, and taste quality among all treatments. In summary, the application of 0.5 kg m^−2^ biochar can improve the biochemical properties of saline soil and therefore increase rice yield.

## 1 Introduction

Salinized land in the world is about 1.7 × 10^9^ ha [1], including nearly 100 million hectares in China, of which one-third has a development potential [2]. To ensure food security, it has become one of strategic development goals in many countries to utilize saline-alkali lands for growing crops, therefore, desalinization and saline soil have received a great attention. Traditional methods of saline-alkali land improvement are mainly through lifting soil bed, salt leaching, digging drainage ditches, setting up underground porous salt discharge pipes, planting rice, and applying soil conditioner, etc. Biochar is a new type of soil amendment and has been widely used for water treatment, soil heavy metal depollution and other fields due to its microporous structure, large specific surface area, high surface energy activity and good adsorption characteristics. In recent years, biochar has also been used to improve saline-alkali soil [3]. Adding biochar to various types of soil can not only increase the availability of key minerals as nutrients, but also amend soil features such as the soil bulk density, hydraulic properties, aggregate structure, ion exchange capacity and microbial activity, so as to make it more conducive for plant growth [[4], [5], [6]]. To further understand water and mineral transport characters in association with the maize growth in the coastal saline soil through using biochar in an irrigation setting of switching saline and freshwater alternatively [7], we demonstrated that biochar could alleviate the adverse effect of saline water irrigation and prevent soil re-salinization after salt-leaching with the saline-freshwater alternate irrigation [8]. found that bamboo biochar could improve the germination, growth, yield, salt tolerance and survival rate of okra [3], and suggested that biochar could absorb Na^+^ in irrigated salt water, promote wheat photosynthesis, enhance nutrient absorption, and increase wheat yield and quality in an experiment using straw biochar in saline soil leached with mild salty water.

According to a comparative study on different soil amendments on saline-alkali land by Qu, et. al [9], adding biochar (22.5 t hm^−2^) proved to be better for soil improvement than other organic fertilizers combined with desulfurization gypsum or single application of desulfurization gypsum (37.5 t hm^−2^). With its growing popularity as an important alternative soil conditioner for saline-alkali land, however [10], Meng et. al found that the conductivity and cation exchange capacity with saline soil increased with more biochar added. The contradict findings seem to indicate that there is no consistent conclusion on whether adding biochar to saline soil will have a positive effect or not.

China is one of world's largest rice growing countries with more than 300, 000 ha and a total output of more than 20 billion tons of rice [11]. During the rice cultivation, there is a window when a layer of water is needed to cover the paddle field, which also serves a purpose of suppressing the soil salinity to grow rice in the saline-alkali land [12]. However, rice planted in the saline-alkali land is often affected by saline-alkali stress and previous studies have shown that rice is capable of activating its limited resistance under saline-alkali stress, but when saline-alkali stress exceeds a tolerant threshold, rice plants suffer from a reduced seed germination rate, downsized leaves, wilting, leaf tip yellowing, less number of tillers, delayed tillering, less spikelets, decreased 1000-grain weight, more chalky grains, and possible death [13]. Therefore, some measures aiming at protection of rice plants from potential damages due to severe saline-alkali stress should be taken to raise the salt tolerant threshold in rice plants.

In summary, this study was designed and executed to determine the effect of biochar on the rice growth and soil properties of the paddle field, reveal the efficacy of different biochar amounts on soil biochemical traits, rice yield and quality, and propose the optimal amount of biochar that can be used for growing rice in saline-alkali lands.

## 2 Materials and methods

### 2.1 Materials

This experiment was carried out at the experimental station of Weifang University of Science and Technology in Shouguang City, Shandong Province (36°86′N, 118°73′E) in 2022. The plot is located in the coastal plain area of northern and central Shandong. The terrain is flat, the average annual temperature is 12.7 °C, the annual sunshine hours are 2548.8 h, and the average annual precipitation is 593.8 mm. Most of precipitation occurs during summer when temperature and other weather conditions are conducive for crop growth. The soil used in the experiment was collected from the coastal saline-alkali land in Yangkou Town, Shouguang City, Shandong Province. The silt loam soil with a composition of 5.49 % clay (<0.002 mm per particle), 66.97 % silt (0.002–0.05 mm per particle), and 27.54 % sand (>0.05 mm per particle) was used throughout the experiment. The electrical conductivity (EC) and pH value of the experimental coastal saline soil with a soil/water ratio at 1: 5 were 1200 μS cm^−1^ and 8.12, respectively. The hydrolyzable nitrogen, available phosphorus, available potassium and organic matter content were 72.9 mg kg^−1^, 4.5 mg kg^−1^, 98 mg kg^−1^ and 7.44 g kg^−1^, respectively. The bulk density of the filled saline soil in barrels was 1.50 g cm^−3^. The test biochar was purchased from Pingdingshan Lvzhiyuan Activated Carbon Co., Ltd. The biochar material was obtained from remnants of maize, wheat, and peanut shells that was pyrolyzed at 600 °C and crushed into 50–100 mesh powder. The total nitrogen, hydrolyzable nitrogen, available phosphorus, available potassium and organic matter content were 1.164 g kg^−1^, 20.4 mg kg^−1^, 665.0 mg kg^−1^, 505.0 mg kg^−1^ and 80.6 g kg^−1^, respectively. The tested rice variety was ‘Tianlongyou 619’.

### 2.2 Experimental design

Six biochar application rates of 0, 0.5, 1, 2, 4 and 6 kg m^−2^ were used with a barrel experimental setting. The height and surface area of the barrel was 38.7 cm and 0.08 m^2^, so the actual amount of biochar in each treatment was 0, 40, 80, 160, 320, 480 g per barrel labeled as CK, T1, T2, T3, T4, T5, with 5 replicates in each treatment. The biochar powder was mixed with a layer of 5 cm top soil, watered and planted with 4 seedlings in each barrel in mid-July 2022. The whole growing season of rice were divided into seedling, jointing, booting, heading, flowering, and filling/maturing stages, during which a layer of 5∼10 cm deep water above soil was maintained through all stages and pesticides sprayed 2–3 times to control pest insects and diseases at the filling stage in the field. Fertilizer was applied once in the jointing stage of rice, using compound fertilizer (N + P_2_O_5_+K_2_O ≥ 50 %, 30-10-10 + TE) at an amount of 2.4 g per barrel.

### 2.3 Sampling and determination methods

#### 2.3.1 Soil index

Soil samples were collected after harvesting rice. Two soil samples at a depth of 0–20 cm were taken from each barrel using a soil drilling sampler and then divided into subsamples as from 0 to 10 cm or 10–20 cm deep soil. A total of 60 soil samples were collected. All soil samples were ground through a sieve with a pore size of 1 mm after air drying, and then divided into 3 parts. Each of 10 g soil sample was mixed with 50 ml deionized water, stirred for 3 min, and measured for the soil EC and pH with a conductivity meter (Leima DDS-307 A of Shanghai Yidian Scientific Instrument Co., Ltd.) and pH meter (Leima PHS-2F). The content of available potassium, phosphorus, nitrogen, and soil organic matter in soil were determined by the NH_4_OAc extraction-flame photometric method, NaHCO_3_ extraction-molybdenum antimony anti colorimetric method, alkaline hydrolysis diffusion method, and potassium dichromate volumetric method-external heating method, respectively. Soil catalase (CAT), acid phosphatase (ACP) and urease (UE) were determined by the spectrophotometry [14]. The specific operation was carried out according to the instructions of soil enzyme determination kit produced by Suzhou Keming Biotechnology Co., Ltd.

#### 2.3.2 Rice growth index

The rice plant height, panicle length and stem diameter were measured by tape and vernier caliper, and the number of tillers and panicles per plant were recorded. After rice plants were matured, their roots, stems and leaves of each treated plant were separated and placed in an envelope and placed in an oven at 105 °C for 30 min, dried to a constant weight at 85 °C, measured for their dry weight of each individual part, and calculated for the total dry weight.

#### 2.3.3 Yield and quality of rice

Rice yield and its components such as the panicle weight per plant, seed setting rate and 100-grain weight were measured after rice grains were harvested and dried. The rice quality index was determined through grinding rice grains in the LTJM160 milled rice machine and placing 150 g milled rice powder into the SATAKE taste meter to automatically detect the taste value, protein and amylose. Due to the limited rice grain available, no replicates were set for this measurement.

### 2.4 Data statistics and analysis methods

Microsoft Office Excel 2021 application was used for data processing and chart making. SPSS Statistics 26 was used for data processing and correlation analysis. The significance level was 0.05.

## 3 Results and analysis

### 3.1 Effect of different biochar amount on soil EC and pH value

**Fig. 1:**
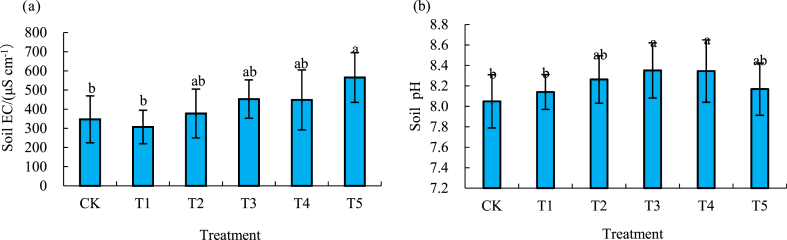
Soil EC (a) and pH (b) in 0–20 cm soil layer under different biochar application rates. The difference between different lowercase letters in the figure represents a significant difference of 0.05. The same notation is followed in the figures below.

With the increase of biochar amount, the mean value of soil EC showed an overall upward trend (Fig. 1). Compared with CK, EC with the T1 treatment decreased by 11.5 %, while EC of the T2 through T5 treatments increased by 8.8 %, 30.5 %, 29.1 % and 62.9 %, respectively. Among them, CK and T1 had a significant lower EC than that of T5, but no significant differences between EC with the rest of treatments (T2-T4) were found (Fig. 1a). The mean value of soil pH increased first and then decreased with more biochar applied. The maximal pH value appeared in T3 and T4 that was significantly higher than that of CK and T1. There were no significant differences between pH values with other treatments. The soil pH of each treatment was weakly alkaline (7 < pH < 8.5) (Fig. 1b).

### 3.2 Effect of different biochar amount on the soil nutrient contents

**Fig. 2:**
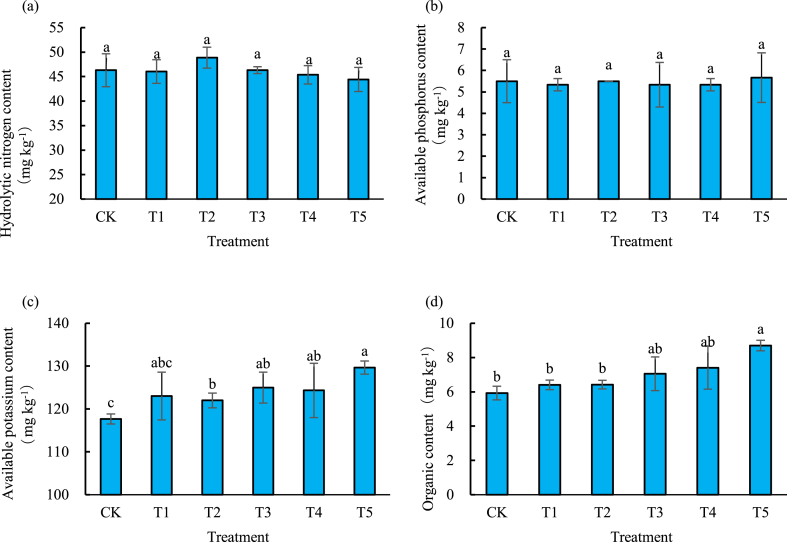
Residual content of hydrolyzable nitrogen (a), available phosphorus (b), available potassium (c) and organic matter (d) in 0–20 cm deep soil with different treatments at various biochar amount.

There were no significant differences of the content of hydrolyzable nitrogen and available phosphorus between different treatments with the increase of the biochar amount [Fig. 2a, b]. However, the content of available potassium and organic matter exhibited an increasing trend. Compared with CK, the content of available potassium in T1-T5 increased by 4.5 %, 3.7 %, 6.2 %, 5.7 % and 10.2 % respectively, and the content of organic matter in those treatments increased by 8.0 %, 8.3 %, 19.1 %, 24.9 % and 46.8 % respectively [Fig. 2c, d].

### 3.3 Effect of different biochar amount on soil enzyme activity

**Fig. 3:**
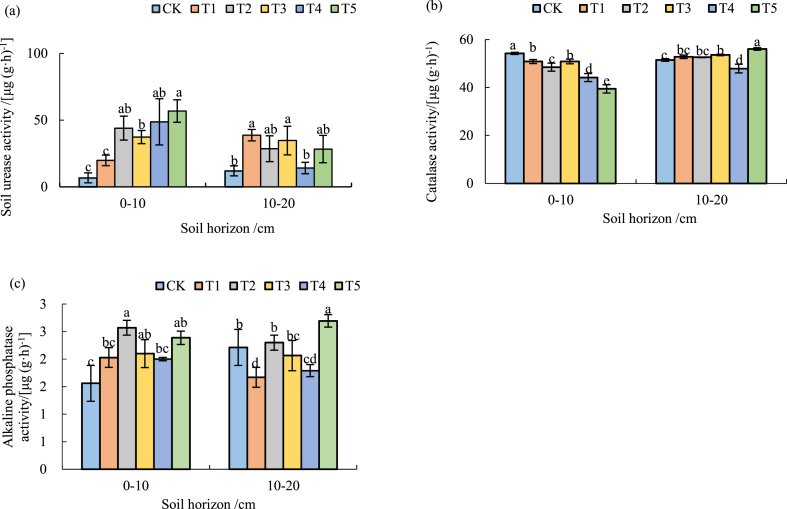
Soil urease (a), catalase (b) and alkaline phosphatase (c) activities in 0–10 and 10–20 cm deep soil at various biochar amount.

The soil urease activity was different at the different depth of soil with different biochar amount (Fig. 3). With the increase of biochar amount, the soil urease activity in 0–10 cm deep soil showed an increasing trend. Compared with CK, T1-T5 had an increased soil urease activity by 194.8 %, 554.1 %, 454.5 %, 623.4 % and 744.6 %, respectively, while that of each individual treatment in 10–20 cm deep soil increased first and then decreased (Fig. 3a). With more biochar amount added, the soil catalase activity in 0–10 cm deep soil decreased. Compared with CK, T1-T5 had a decreased soil catalase activity of 6.3 %, 10.6 %, 6.3 %, 18.6 % and 27.3 %, respectively. The soil catalase activity in 10–20 cm deep soil varied with the increase of biochar amount but significantly (Fig. 3b). With the increase of biochar amount, the soil alkaline phosphatase activity in 0–10 cm deep soil increased first and then decreased. Compared with CK, T1-T5 had an increased soil alkaline phosphatase activity of 30 %, 64.8 %, 34.7 %, 28.2 % and 53.1 % respectively. There was a significant difference in 10–20 cm deep soil among treatments, especially with T5, but the variation seemed to be random without a uniform pattern (Fig. 3c).

### 3.4 Effect of different biochar amount on rice growth

There was no significant difference in the rice plant height, stem diameter, tiller number, panicle length and single panicle weight between the T1-T5 and CK treatments (Table 1). The total root length of rice increased first and then decreased with the increase of biochar amount. In comparison with the CK treatment, T1 and T2 treatment had an increased total root length by 33.9 % and 11.3 %, respectively, and the T3-T5 treatments had a decreased one by 7.3 %, 29.7 % and 38.6 %, respectively (see Table 2).

**Table 1:** Effect of different biochar amount on rice growth index.

Treatment	Plant height（cm）	Stem diameter（mm）	Total root length（cm）	Tiller number	Spike length（cm）	Grain mass per panicle（g）
CK	68.54 ± 3.56 ab	3.34 ± 0.88a	700 ± 125bc	5 ± 0.68 ab	12.95 ± 0.98 ab	1.46 ± 0.42 ab
T1	71.26 ± 2.18a	4.03 ± 0.71a	937 ± 162a	5 ± 0.77 ab	14.01 ± 0.45a	1.70 ± 0.21a
T2	64.59 ± 4.53bc	3.57 ± 0.61a	779 ± 62 ab	4 ± 1.21 b	13.33 ± 0.75 ab	1.50 ± 0.24 ab
T3	68.66 ± 3.75 ab	3.55 ± 0.78a	649 ± 151bcd	5 ± 0.75 ab	13.59 ± 0.47 ab	1.47 ± 0.09 ab
T4	61.54 ± 3.92c	3.51 ± 0.44a	492 ± 215cd	4 ± 0.79 ab	13.05 ± 0.42 ab	1.14 ± 0.23 b
T5	65.08 ± 5.31bc	3.89 ± 0.66a	430 ± 130 d	6 ± 0.62a	12.62 ± 0.92 b	1.39 ± 0.22 ab

Note: The difference between different lowercase letters under the same column number in the table represents a significant difference of 0.05.

**Table 2:** Rice quality indexes of different treatments.

Treatment	Milling（%）	Taste value	Protein（%）	Amylose（%）
CK	64.3	57	9.1	20.8
T1	66.6	56	9.4	21.7
T2	67.5	58	9.2	20.7
T3	68.4	58	9.0	20.6
T4	67.0	59	8.8	20.5
T5	67.5	57	9.3	20.8

Note: Due to a limited grain yield of rice with each treatment, the measurement was not repeated.

### 3.5 Effect of different biochar amount on rice dry matter quality

**Fig. 4:**
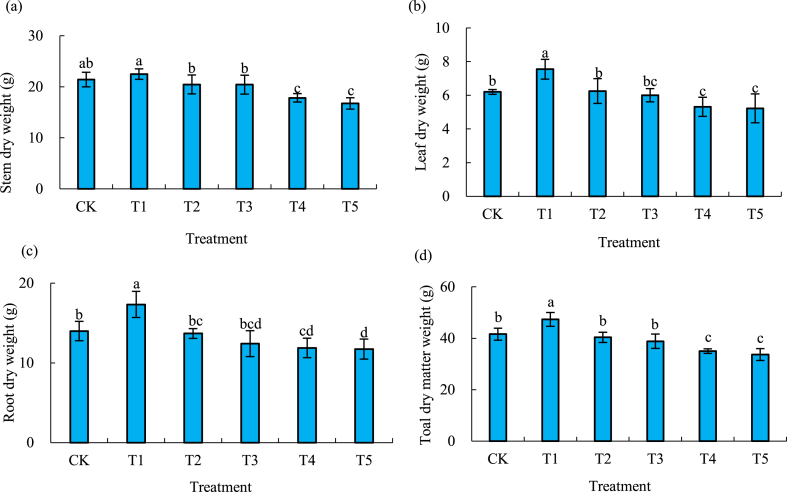
Stem dry weight (a), leaf dry weight (b), root dry weight (c) and total dry matter weight (d) of rice with different biochar amounts.

With the increase of biochar amount, the quality of rice stems, leaves, roots and total dry matter increased first and then decreased and the maximal values appeared in T1 (Fig. 4). Compared with no biochar (CK), the dry weight of rice stems of T1 increased by 5 %, while that with T2, T3, T4 and T5 decreased by 4.5 %, 4.7 %, 16.8 % and 21.8 %, respectively (Fig. 4a). Compared with CK, the dry weight of rice leaves of T1 and T2 increased by 21.8 % and 0.8 %, respectively, while that with T3, T4 and T5 decreased by 3.1 %, 14.3 % and 15.8 %, respectively (Fig. 4b). The dry weight of rice roots of T1 increased by 23.9 %, while that of T2, T3, T4 and T5 decreased by 2.2 %, 11.2 %, 15.1 % and 16.1 %, respectively in comparison with that of CK (Fig. 4c). Compared with CK, the total dry matter weight of rice of T1 increased by 13.8 %, while that of T2, T3, T4 and T5 decreased by 2.9 %, 6.6 %, 15.8 % and 19.0 %, respectively (Fig. 4d).

### 3.6 Effect of different biochar amount on rice yield and its components

**Fig. 5:**
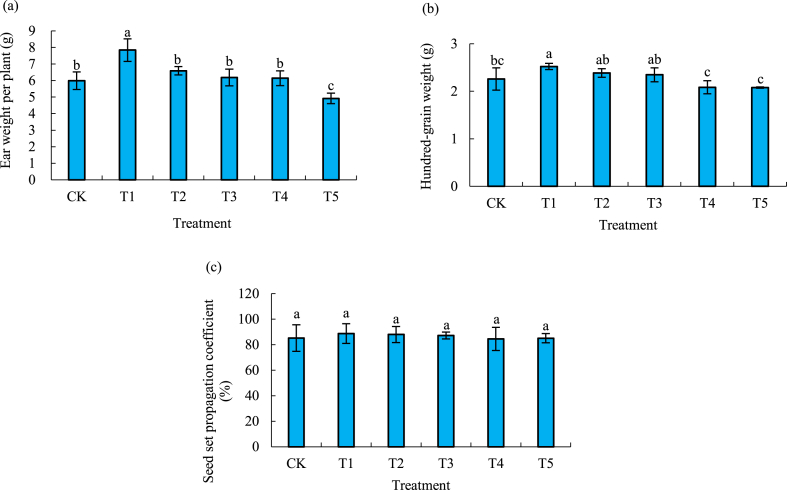
Panicle weight per plant (a), seed setting rate (b) and 100-grain weight (c) of rice with different biochar amounts.

With the increase of biochar amounts, the panicle weight per plant in general increased first and then decreased (Fig. 5a) in comparison with that of CK, while that of T2, T3 and T4 did not increase significantly, except that of T1 with an increase by 30.8 % and that of T5 decreased by 17.9 %. The 100-grain weight of T1 significantly increased by 11.6 % more than that of CK and other treatments (Fig. 5b). Biochar did not seem to have any effects on the seed setting rate (Fig. 5c).

### 3.7 Effect of different biochar amount on rice quality

In comparison with the CK treatment, T1-T5 treatments yielded slightly more by 3.6–6.4 %, while the rice taste, protein and amylose content remained unchanged. Therefore, no effect of biochar on rice grain quality was observed.

## 4 Discussion

### 4.1 Effect of adding various biochar amount to saline soil on chemical properties and nutrient content

In general, adding biochar to amend soil of our experimental sites increased the soil pH and EC value, which was consistent with findings reported by Meng et. al. [10]. The increase of soil pH might be due to the alkaline substance in biochar itself, while the strong adsorption capacity of biochar may have kept salts in water. In addition, more available potassium and organic matter instead of hydrolyzable nitrogen and available phosphorus were found in the soil amended with biochar. It seems that straw biochar is rich in potassium itself and its porous structure provides an ideal habitat for potassium-solubilizing microbes to accumulate potassium [15]. The accumulation of soil organic matter is because: 1) biochar itself contains abundant organic matter; 2) biochar has abundant pore structure and large specific area to adsorb and retain active organic matters; and 3) biochar inhibits soil respiration and reduced the mineralization rate of soil organic carbon [16]. Also, the high carbon nitrogen ratio and adsorption of biochar may have kept the hydrolyzable nitrogen content unchanged in soil [17] and the unchanged content of phosphorus may be due to the fact that soil is prone to retain it readily.

### 4.2 Effect of adding biochar to saline soil on soil enzyme activities

Soil enzyme activities reflect soil fertility and are an important part of soil biomes [18]. The soil urease directly participates in the transformation of nitrogen-containing organic matter to N available to plants in the soil, and its active level is an indicator of the soil nitrogen level [19], involving in the redox reaction beneficial to plant metabolism [20], while the soil phosphate activity indicated the transformation level of organic phosphorus in soil [21]. This study showed that the soil urease activity of each biochar treatment in 0–20 cm deep soil was higher than that of CK, and so was the soil alkaline phosphatase activity in 0–10 cm deep soil, which is consistent with the report by Gu, et. al. [22] in which adding biochar has promoted the activity of urease and soil alkaline phosphatase in saline-alkali soil. With the increase of biochar amount added, the soil catalase in 0–10 cm deep soil decreased possibly due to the strong adsorption of biochar, resulting in soil catalases being adsorbed and contained to react with other substances [23] [24]. In addition, the overall activities of soil enzymes at different soil layers and between various biochar treatments vary with the amount added, which is also common in previous studies.

### 4.3 Effect of adding biochar to saline soil on rice growth and development

A previous study has shown that applying an appropriate amount of biochar can increase crop yield [14] and our findings with this study have further demonstrated that an appropriate amount of biochar added to soil promoted the rice growth and development through promoting rice plant height, root lengths, dry matter amount and yield. The underline mechanisms of biochar involved in enhancement of plant growth may include: 1) the soil EC is lower with an appropriate amount of biochar applied in soil to alleviate rice salt stress; 2) the soil porosity increased with an addition of biochar that is conducive to the microbial catalysis and activities pertaining to the soil physical and chemical reactions for improvement of root surrounding environment and the root growth; and 3) biochar itself contains nutrients such as nitrogen, phosphorus, potassium and calcium, magnesium, zinc and other trace elements required for rice that are needed to overcome salt stress [25]; [26]; [18]. Biochar is loose and porous to absorb and retain nutrients and water and promote accumulation of dry matter in rice plants [4]. However, excessive amount of biochar may interfere with a normal plant growth since it also contains some substances that are inhibitory to the plant growth [27]. An application of 0.5 kg m^−2^ biochar in the experiment should be enough to increase the plant height and root length of rice under saline-alkali soil, and its excessive application more than that amount will have a negative effect, which is different from the amount of 1.65 kg m^−2^ biochar recommended by [28] for soda plants grown in saline-alkali, possibly due to the differences in the soil chemical compositions, plant type and biochar properties.

### 4.4 Effect of adding biochar to saline soil on rice quality

Niu, et. al. [29] found that the use of biochar had no significant effect on the overall protein content and amylose content but the taste of rice grains was significantly improved. In addition [18], Guo, et. al. demonstrated that biochar did not affect the amylose content in rice, but significantly increased the yield and protein content. Therefore, the effect of using biochar to improve rice quality varies mainly because the rice quality is affected by many factors such as local climatic conditions, nutritional compositions and relevant gene expressions. This study has confirmed that biochar slightly increased the rice yield, but insignificantly affected the rice taste, protein levels and amylose contents. So far, there are few studies focused on explore how biochar affects quality of rice grains produced from saline soil and more thorough and well-designed researches are needed through long-term experiments to fully understand the functions of biochar in affecting rice quality and overall content.

## 5 Conclusion

This study has demonstrated that adding biochar to saline soil can effectively increase the activity of soil ureases and soil alkaline phosphatases that are required to make soil potassium and organic matter more available, which promotes the rice plant height, root length and dry matter, and ultimately increase the rice yield. For an effective and efficient use to amend the biochemical properties of soil and increase rice yield, biochar should be applied at a 0.5 kg m^−2^ rate and mixed evenly with 5 cm thick surface saline soil, which can provide reference for rice production in saline-alkali land. The future research to follow this study should focus on the effect of biochar on the dynamics of soil microbiome and its storage and transformation in soil to provide an overall, theoretical and practical use of biochar for sustainable agriculture.

## Data availability statement

Data will be made available on request.

## CRediT authorship contribution statement

**Shuqian Zhang:** Writing – original draft, Data curation. **Lingyue Wang:** Writing – original draft, Investigation, Data curation. **Junping Gao:** Writing – original draft, Methodology, Data curation. **Baichuan Zhou:** Software, Investigation. **Wei Hao:** Resources, Methodology. **Di Feng:** W. **Xiaoan Sun:** Writing – review & editing, Validation, Supervision.

## Declaration of competing interest

The authors declare that they have no known competing financial interests or personal relationships that could have appeared to influence the work reported in this paper.
